# Immunomodulation for primary prevention of urinary tract infections in patients with spinal cord injury during primary rehabilitation: protocol for a randomized placebo-controlled pilot trial (UROVAXOM-pilot)

**DOI:** 10.1186/s13063-021-05630-w

**Published:** 2021-10-04

**Authors:** Jörg Krebs, Jivko Stoyanov, Jens Wöllner, Ezra Valido, Jürgen Pannek

**Affiliations:** 1 Clinical Trial Unit, Swiss Paraplegic Centre, Nottwil, Switzerland; 2 SCI Population Biobanking & Translational Medicine Group, Swiss Paraplegic Research, Nottwil, Switzerland; 3 Institute of Social and Preventive Medicine (ISPM), University of Bern, Bern, Switzerland; 4 Neuro-Urology, Swiss Paraplegic Centre, Nottwil, Switzerland; 5 Department of Urology, Inselspital, Bern University Hospital, University of Bern, Bern, Switzerland

**Keywords:** Spinal cord injury, Neurogenic lower urinary tract dysfunction, Urinary tract infection, *Escherichia coli*, Uro-Vaxom®, Immunotherapy, Immunomodulation, Immunostimulation, Feasibility trial, SwiSCI, SwiSCI biobank

## Abstract

**Background:**

Urinary tract infections (UTIs) are common in individuals with neurogenic lower urinary tract dysfunction (NLUTD) following spinal cord injury (SCI). They are not only a great burden for affected individuals, but also cause considerable health costs. Furthermore, recurrent antibiotic treatments of UTIs contribute to the growing problem of bacterial resistance to antimicrobial compounds. Even though there is a multitude of different measures to prevent UTIs in individuals with NLUTD, no clear evidence exists for any of these. Oral immunomodulation with UTI-relevant *Escherichia coli* lysate may be a promising preventative measure with a good safety profile in individuals with NLUTD. However, currently available data are sparse.

**Methods:**

This is a randomized, quasi-blinded, placebo-controlled, mono-centric pilot trial investigating the feasibility of a main trial regarding the effects of a lyophilized lysate of *E. coli* strains for oral application (Uro-Vaxom®, OM Pharma SA, Meyrin, Switzerland). There will be two parallel groups of 12 participants each. Individuals with acute SCI (duration SCI ≤ 56 days) from 18 to 70 years of age admitted for primary rehabilitation will be eligible. Blood and urine samples will be taken prior to intervention start, at the end of the intervention, and 3 months after intervention termination. The trial intervention will last 90 days. The participants will not be informed regarding the treatment allocation (quasi-blinded). The nursing staff will prepare the daily dose of the allocated treatment from the original packaging. The trial personnel and the biostatistician will be blinded. Feasibility (e.g., recruitment rate, patient attrition), clinical (e.g., number of symptomatic UTIs), and laboratory parameters (e.g., urinary culture, urinary proteo- and microbiome, blood cell counts) as well as adverse events will be collected.

**Discussion:**

Effective and efficient measures for the prevention of UTIs in individuals with NLUTD are urgently needed. If the conclusion of this pilot is positive regarding feasibility, the effects of oral immunomodulation with a *E. coli* lysate will be investigated in a larger, sufficiently powered, multi-center trial.

**Trial registration:**

ClinicalTrials.govNCT04049994. Registered on 8 August 2019

**Supplementary Information:**

The online version contains supplementary material available at 10.1186/s13063-021-05630-w.

## Administrative information

Note: the numbers in curly brackets in this protocol refer to SPIRIT checklist item numbers. The order of the items has been modified to group similar items (see http://www.equator-network.org/reporting-guidelines/spirit-2013-statement-defining-standard-protocol-items-for-clinical-trials/).

**Table:** 

Title {1}	Immunomodulation for primary prevention of urinary tract infections in patients with spinal cord injury during first rehabilitation: protocol for a randomized controlled pilot trial (UROVAXOM-Pilot)
Trial registration {2a and 2b}	ClinicalTrials.gov identifier: NCT04049994 registered 8 August 2019
Protocol version {3}	Version 1.3 July 7, 2020
Funding {4}	Nested project start-up grant from the Swiss Spinal Cord Injury Cohort Study (SwiSCI) and the Swiss Paraplegic Foundation
Author details {5a} Jörg Krebs Aff1 Jivko Stoyanov Aff 2 & 3 Jens Wöllner Aff 4 Ezra Valido Aff 2 Jürgen Pannek Aff 4 & 5	1: Clinical Trial Unit, Swiss Paraplegic Centre, Nottwil, Switzerland; 2: SCI Population Biobanking & Translational Medicine Group, Swiss Paraplegic Research, Nottwil, Switzerland; 3: Institute of Social and Preventive Medicine (ISPM), University of Bern, Bern, Switzerland; 4: Neuro-Urology, Swiss Paraplegic Centre, Nottwil, Switzerland; 5: Department of Urology, Inselspital, Bern University Hospital, University of Bern, Bern, Switzerland
Name and contact information for the trial sponsor {5b}	Swiss Paraplegic Foundation, Guido A. Zäch Street 10, CH-6207 Nottwil, Switzerland Swiss Paraplegic Research, Guido A. Zäch Street 4, CH-6207 Nottwil, Switzerland
Role of sponsor {5c}	The sponsors and funders mentioned above have no role in nor any authority over study design; collection, management, analysis, and interpretation of data; writing of the report; and the decision to submit the report for publication.

## Introduction

### Background and rationale {6a}

Urinary tract infections (UTIs) continue to be one of the most common secondary complications in individuals with neurogenic lower urinary tract dysfunction (NLUTD) as a result of spinal cord injury (SCI) [1, 2]. The main contributors to the increased risk of UTI in these individuals are impaired urine storage and voiding function, the use of catheters, and a premature onset of immuno-depression [3–6]. Recurrent UTIs do not only entail considerable health costs, but also decreased quality of life [7, 8]. Furthermore, increased bacterial resistance to antimicrobial compounds as a result of repeated antibiotic treatments of UTIs is a major concern [9].

Apart from optimizing lower urinary tract function and removing morphologic causes of infection (i.e., bladder stones) [10], different remedies, such as phytotherapeutics, urine acidifiers, and antiseptic substances, are frequently applied in order to prevent UTIs in individuals with NLUTD [5]. However, there is still no clear evidence for an effect of these therapies [10]. In women without NLUTD, oral immunomodulation with a lysate of UTI-relevant *Escherichia coli* appears to be effective in the prevention of UTIs. The intervention resulted in a significantly lower UTI rate and use of antibiotics compared to placebo [11, 12]. Immunomodulation with *E. coli* fractions may also be a promising preventative measure in individuals with NLUTD. However, currently available data are sparse and limited as a result of evaluating clinically not relevant outcome measures (i.e., presence of bacteriuria) [13] or the nature of the investigations (i.e., retrospective [14]/feasibility study [15]). Oral immunomodulation with a *E. coli* lysate shows a good safety profile [11, 12, 15], and thus, its efficacy in individuals with NLUTD should be investigated.

## Objectives {7}

The primary objective of this pilot trial is to evaluate the feasibility of a main trial. The secondary objective is to collect data for an informed sample size calculation. A further objective is to investigate the clinical (number of symptomatic UTI), biological (stimulation of the immune system), and urinary microbiome changes after immunomodulation.

## Trial design {8}

This is a randomized, placebo-controlled, mono-centric pilot trial with two parallel groups of 12 trial participants each (1:1 allocation). Allocation will be determined by block-randomization stratified according to sex. The reason for stratification according to sex is the higher risk of UTIs in women with SCI [16]. Trial participants will receive the intervention for 90 days and will be followed up for 3 months thereafter.

## Methods: participants, interventions, and outcomes

### Study setting {9}

The trial is embedded within the Swiss Spinal Cord Injury Cohort Study (SwiSCI) as a Nested Project and benefits from the services of the SwiSCI Biobank. We decided to run this trial in a single center, because the primary objective is to evaluate the feasibility of a main trial. The trial center is with 206 beds the largest rehabilitation and acute care center specialized in SCI in Switzerland.

### Eligibility criteria {10}

Individuals with acute SCI (duration SCI ≤ 56 days) from 18 to 70 years of age during primary rehabilitation (hospitalization at least until termination of trial intervention) are eligible for the trial. The onset of SCI has to have occurred within 72 h. Eligible patients need to consent to participation in the SwiSCI cohort study prior to consenting to the present trial. The presence of any one of the following criteria will lead to the exclusion of an individual or trial participant:

- Bladder evacuation by permanent transurethral catheterization at the trial start
- Known hypersensitivity to investigational product or placebo (galactose, fructose, lactose)
- Any other therapies for preventing UTIs (e.g., urine acidification, phytotherapy)
- Immunomodulation (apart from routine vaccinations)
- Immunosuppressant therapy
- Oncological or autoimmune disease
- Diabetes mellitus, nephropathy, bladder stones
- Chronic bacterial prostatitis, recurrent UTIs prior to SCI
- Women who are pregnant or breastfeeding
- Drug or alcohol abuse
- Suspected inability to follow the procedures of the trial (e.g., language problems, psychological disorders, dementia)
- Participation in an interventional trial affecting the urinary tract or immune system during and within the 30 days preceding the present trial

### Who will take informed consent? {26a}

Investigators who are board-certified neuro-urologists will inform eligible patients about the trial and request them to read the trial information. The ethics committee of Northwestern and Central Switzerland (Project ID 2019-01768; final approval 26.11.2019) has approved the written trial information and consent form. The written informed consent will be obtained not earlier than 24h after the initial oral information. Eligible patients will have the opportunity to ask questions regarding the trial. There will be no monetary or other compensation for trial participation.

### Additional consent provisions for collection and use of participant data and biological specimens {26b}

Potential trial participants will be asked to give additional consent for the use of the personal health data and biological specimens collected during the trial in future clinical research projects.

## Interventions

### Explanation for the choice of comparators {6b}

The active intervention has been approved in Switzerland for the prevention of recurrent infections of the lower urinary tract and as an auxiliary treatment of acute UTIs. Placebo is the standard comparator in clinical trials investigating the effect of a medicinal product, if placebo treatment is ethically justifiable. In the present trial, participants allocated to the placebo group will not incur any disadvantages, because the trial protocol does not prevent standard clinical treatment of UTIs with antibiotics. The effectiveness of Uro-Vaxom® has not yet been established in individuals with SCI, and participants allocated to the placebo group may receive Uro-Vaxom® treatment after trial termination.

### Intervention description {11a}

#### Experimental group

Uro-Vaxom® (OM Pharma SA, Meyrin, Switzerland) is a lyophilized lysate of 18 *E. coli* strains (6 mg) for oral application. The treatment will last 90 days (one capsule daily). The dose and duration of the experimental treatment have been chosen according to the medicinal product package leaflet.

#### Control group

Off-the-shelf placebo tablets (P-Dragees, Zentiva Pharma GmbH, Germany) will be used as control treatment. According to the treatment regimen of the experimental group, trial participants in the control group will receive one sugar-coated tablet daily for 90 days.

### Criteria for discontinuing or modifying allocated interventions {11b}

Allocated treatments will not be modified. The criteria for discontinuation of the interventions are informed consent withdrawal, an intervention-related serious adverse event (SAE), or the presence of a listed exclusion criterion.

### Strategies to improve adherence to interventions {11c}

In-house patients during primary rehabilitation will be enrolled. Trial participants will remain hospitalized for the entire duration of the trial intervention (see eligibility criteria). The nursing staff will prepare the trial drug or placebo daily and monitor the ingestion. Any failure to ingest the trial drug or placebo will be documented and reported to the principal investigator.

The trial coordinator will monitor the reporting and documentation of UTIs (symptoms, laboratory results) and their treatment in trial-specific case report forms (CRFs). If trial participants get discharged from primary rehabilitation prior to trial termination and do not return the CRFs regarding UTIs, the trial coordinator will collect the required data during telephone interviews.

### Relevant concomitant care permitted or prohibited during the trial {11d}

Standard clinical treatment of acute UTIs (i.e., antibiotics, bladder irrigation) will be permitted. However, other measures to prevent the occurrence of UTIs (e.g., urine acidification or phytotherapy) will not be permitted. According to evidence-based medicine criteria, none of the currently applied prophylactic measures can be recommended.

### Provisions for post-trial care {30}

There are no provisions for post-trial care, as there is no risk of harm as a result of the trial intervention after intervention termination and beyond follow-up period.

### Outcomes {12}

#### Primary outcome

The randomization rate will be determined by calculating the proportion of eligible patients who are enrolled and randomized during the trial.

#### Secondary outcomes: feasibility

- Positive screening rate: proportion of screened patients who are eligible
- Recruitment rate: proportion of eligible patients who gave consent
- Treatment-specific retention rates: proportion of randomized trial participants in each treatment arm who finish the trial
- Treatment-specific adherence rate: proportion of trial participants allocated to a treatment arm who complete (≥60 capsules/tablets taken) the treatment
- Reasons for premature trial termination
- Assessment completion rate: proportion of planned assessments that are completed

#### Secondary outcomes: clinical measures

- UTI symptoms during the treatment and the follow-up period: self-report questionnaire completed fortnightly (new/increased incontinence/leaking or increased urgency or increased catheterization frequency, fever/chills, increase in spasticity, malaise or feeling sick or fatigue, cloudy and foul-smelling urine, bladder or lower back pain or pain during urination, new/increased occurrence of goosebumps or sweating)
- Urine analysis (at baseline, treatment end, trial end, and if clinically indicated): leucocytes, nitrite, pH, protein, blood determined by dip stick, and leucocyte count in sediment
- Urine culture results (if clinically indicated): bacterial species, colony-forming units (cfu), and antibiotic resistance
- UTI count: an event is counted as a UTI if ≥10,000 cfu/ml and a positive urine analysis result (leucocytes, nitrite) are present
- Urinary microbiome (at baseline, treatment end, trial end)
- Urinary proteome/transcriptome (at baseline, treatment end, trial end): cytokines, immunoglobulin (Ig)A levels
- Blood cell count (at baseline, treatment end, trial end): leucocytes, erythrocytes, hemoglobin, hematocrit, mean corpuscular volume, mean corpuscular hemoglobin, mean corpuscular hemoglobin volume, and platelets
- White blood cell differential (at baseline, treatment end, trial end): granulocytes, neutrophils, eosinophils, basophils, monocytes, and lymphocytes
- Side effects: self-report questionnaire completed fortnightly during the treatment period

#### Other outcomes of interest

- Patient characteristics: age, sex, lesion level, American Spinal Injury Association impairment scale (AIS), duration SCI, and etiology SCI
- Bladder evacuation method
- Concurrent medication
- Medical history: infections, urinary tract disease, and surgery
- Self-report questionnaire trial end: trial experience (burden trial medication, burden questionnaire completion, “would you participate again?,” would you recommend trial participation?”), satisfaction with treatment effect and assumption regarding treatment allocation

### Participant timeline {13}

**Fig. 1:**
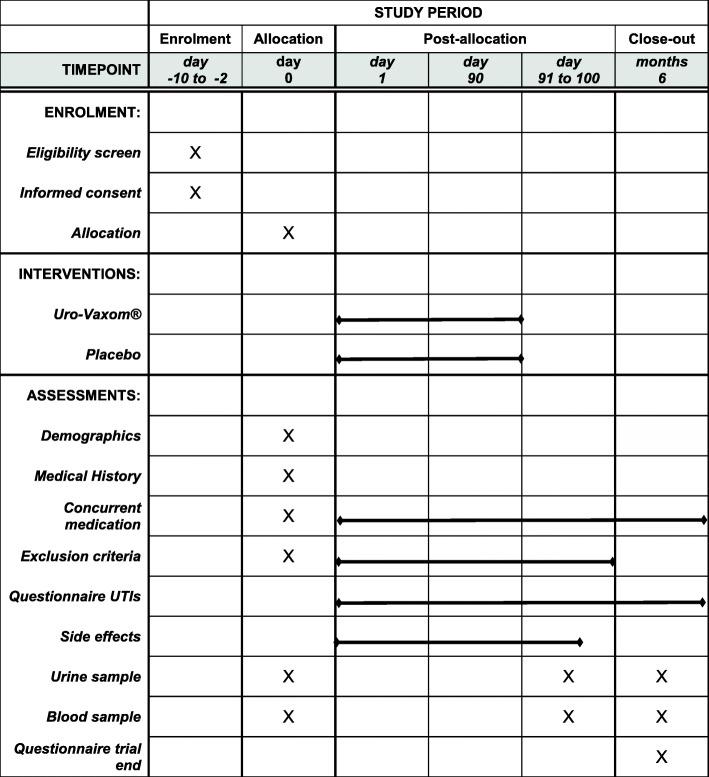
Schedule of enrolment, interventions, and assessments. UTIs urinary tract infections

The schedule of enrolment, interventions, assessments, and visits for participants is shown in Fig. 1. Eligible patients will be approached regarding trial participation after admission for primary rehabilitation as soon as their physical and mental condition allows. The trial intervention will commence not later than 56 days after SCI has occurred. Prior to intervention start, blood and urine samples will be taken as well as at the end of the intervention and 3 months after intervention termination (Fig. 1). The trial intervention will last 90 days. During the intervention and follow-up period (3 months), the occurrence of UTIs, symptoms, and treatment will be recorded. At the trial end, participants will fill out a questionnaire regarding their experiences during the course of the trial. In participants who prematurely stop the trial, a safety follow-up period of 7 days will be observed to monitor the occurrence of SAEs.

### Sample size {14}

A total of 24 participants will be included based on the rule of thumb of 12 participants per treatment arm in pilot studies suggested by Julious and Owen [17]. The sample size will be sufficient to evaluate the feasibility of a main trial which is the primary objective of this pilot trial. If more than 6 participants in a treatment arm terminate the trial prematurely, additional participants will be recruited until at least 6 have completed the trial protocol.

### Recruitment {15}

All patients with acute SCI admitted for primary rehabilitation will be screened for inclusion and exclusion criteria by authorized trial staff.

## Assignment of interventions: allocation

### Sequence generation {16a}

The allocation sequence was generated stratified according to sex, with a 1:1 allocation ratio and variable block sizes using the R software environment (version 3.6.0, Copyright 2019, The R Foundation for Statistical Computing) and the package “blockrand.” The allocation list contains 20 male and 12 female participants.

### Concealment mechanism {16b}

The allocation list has been imported into the trial database. Upon registration of a new trial participant in the database, a sequential number will be allocated for each stratum separately. The hospital pharmacy keeps the list linking the allocation number with the treatment arm (A or B) and will deliver the allocated treatment to the ward. The trial personnel has no access to the list. The biostatistician will receive the blinded treatment allocation (A or B) list when data collection will have been completed. Furthermore, the laboratory personnel will be informed concerning the blinded treatment allocation, in order to ensure balance between allocation groups for the analysis of a specific batch of samples and thus avoid a batch effect.

### Implementation {16c}

A biostatistician of the Swiss Paraplegic Centre Clinical Trial Unit (CTU) has generated the allocation sequence. Authorized trial personnel will register participants in the trial database and inform the head of the hospital pharmacy regarding the allocation number. The head of the pharmacy will assign participants to treatments based on the allocation list.

## Assignment of interventions: blinding

### Who will be blinded {17a}

The trial interventions (Uro-Vaxom® and placebo) will be delivered to the ward within the original packaging supplied by the hospital pharmacy. The trial medication will be personalized and labeled for each study participant. The nursing staff will daily prepare a single dose of the allocated treatment (removing capsule or tablet from blister or container). Preparation and administration of the trial medication will follow the study working instruction, will be overseen by nursing experts, and will be recorded in the hospital information system. Trial participants will not be informed regarding the treatment allocation. The trial treatment will be prescribed as “Uro-Vax trial medication” in the electronic medical records system. The trial personnel (outcome assessors) and biostatistician will be blinded regarding treatment allocation.

### Procedure for unblinding if needed {17b}

Unblinding will be permissible in the occurrence of a SAE. A code break is not required because the nursing staff is not blinded to the treatment arm. Furthermore, unblinding will be performed after having completed all data analyses.

## Data collection and management

### Plans for assessment and collection of outcomes {18a}

#### Feasibility outcomes

An electronic list (Microsoft Excel 2016) of all screened patients will be maintained, and information regarding eligibility, exclusion criteria, and enrolment will be collected. Additionally, a paper (p)CRF regarding all inclusion and exclusion criteria will be completed before enrolment. Exclusion criteria will also be recorded on a pCRF fortnightly during the intervention period as well as at the end of the intervention and follow-up period. Any premature trial termination and the reasons will be documented in an electronic (e)CRF of the trial database. Furthermore, the number of ingested trial medication (capsules/tablets) will be documented in an eCRF.

#### Clinical outcomes

During the intervention period, trial participants will complete a self-developed paper questionnaire regarding UTI symptoms and treatment side effects every 2 weeks. The following UTI symptoms are listed: fever/shivering; new or increased urinary incontinence/leaking, increased urgency, increased catheterization frequency; increased spasticity; malaise, feeling sick, fatigue; cloudy, foul-smelling urine; abdominal/lower back/kidney pain, pain during bladder voiding; and goose bumps, increased sweating. Participants have the possibility to list any other symptoms experienced. An event is counted as a symptomatic UTI if at least one bladder symptom (new or increased urinary incontinence/leaking, increased urgency, increased catheterization frequency, pain during bladder voiding) and another symptom have been reported together with a positive urine analysis result (leucocytes, nitrite) and a growth of ≥10,000 cfu/ml in the urine culture (sample collected by *via naturalis*).

The following side effects are listed: headache, abdominal pain, nausea, vomiting, diarrhea, and heartburn. Participants have the possibility to list any other side effects experienced. In the event of a symptomatic UTI, data from clinical routine assessments (i.e., urinary status and urine culture results) and the chosen treatment will be collected from the electronic medical chart file and recorded in an eCRF.

During the follow-up period, trial participants will complete a self-developed paper questionnaire regarding UTI symptoms (same as above), assessments (dip stick and urine culture), and treatment fortnightly. In the event of a symptomatic UTI in still hospitalized trial participants, data from clinical routine assessments (i.e., urinary status and urine culture results) and the chosen treatment will be collected from the electronic medical chart file and recorded in an eCRF.

#### Biological outcomes

At the three assessment time points (baseline, treatment end, trial end), urine (30ml) and blood samples (30ml) will be taken. The urinary status, proteome and transcriptome, microbiome, and the standard blood cell count and white blood cell differential will be evaluated.

In order to assess the urinary microbiome, urine will be cultured using a streamlined enhanced quantitative urine culture described by Price et al. [18]. Microbial identification will be performed using matrix-assisted laser desorption/ionization–time-of-flight mass spectrometry (MALDI-TOF MS; Bruker Daltonics, Billerica, MA, USA) of distinct colonies. The colony morphology will be described and quantified. Furthermore, within 4h of collection, urine will be spun at 3000rpm, and the resulting pellet will be suspended in 1ml urine for storage at − 80°C until deoxyribonucleic acid (DNA) extraction. The DNA extraction will be accomplished using the QIAamp PowerFecal DNA kit (QIAGEN, Hilden, Germany) according to the manufacturer’s protocol. Whole genome (sufficient quantity of genomic DNA extracted) or 16S sequencing (smaller quantity of DNA extracted) will be performed using the MinION nanopore sequencer (Oxford Nanopore Technologies, Oxford, UK). Sequencing libraries will be prepared using the Nanopore Ligation Sequencing Kit (Oxford Nanopore, Oxford, UK) and sequenced with the MinION flow cell using a high accuracy model. Bioinformatics analysis will use the FASTQ WIMP (“What’s In My Pot”) (Oxford Nanopore, Oxford, UK) analysis workflow validated for microbiome analysis.

In order to assess the relevant proteins of the urinary proteome and transcriptome, we will use two sample types: (1) centrifuged and sterile filtered (0.22μm) urine and (2) urine pellets (where present). These samples will be analyzed by enzyme-linked immunosorbent assay (ELISA) for total quantity of IgA antibodies and quantity of IgA antibodies against the *E. coli* lysate included in Uro-Vaxom®. The quantity of pro- and anti-inflammatory cytokines such as interferon (IFN)-γ, tumor necrosis factor (TNF)-α, tumor growth factor (TGF)-β, and interleukin (IL)-1β, IL-2, IL-4, IL-5, IL-6, IL-8, IL-10, IL-13 will be determined using ELISA and reverse transcription polymerase chain reaction (RT-PCR) arrays of extracted ribonucleic acid (RNA).

#### Other outcomes of interest

Patient characteristics, bladder evacuation method, and concurrent medication will be collected from the electronic medical chart file and recorded in an eCRF. Data regarding the medical history will be collected using a pCRF. At the end of the follow-up period, participants will fill out a self-developed pCRF regarding their experiences, treatment satisfaction, and assumption regarding treatment allocation.

### Plans to promote participant retention and complete follow-up {18b}

Trial participants will remain hospitalized during the treatment period (see eligibility criteria). If a trial participant is discharged before completion of the trial, telephone interviews will be planned to collect outcome measures regarding UTIs, in case the participant does not return the self-report questionnaires.

In participants who discontinue or deviate from intervention protocols, all outcome data as per protocol will be collected as long as participants stay in the trial. In case of premature trial termination, clinical routine data regarding UTIs will be collected, if participants have not rejected the use of health-related data and samples for research purposes (general consent).

### Data management {19}

Trial source data will be recorded in pCRFs or directly in eCRFs (see above). All data will be recorded encrypted. Trial data from pCRFs will be transferred to eCRFs in a timely manner. The eCRFs will be kept current to reflect the participant’s status during the course of the trial. The eCRFs have been created in the web-based data management system secuTrial® (iAS, Berlin, Germany). The system is hosted on a secure in-house server. Only trained and authorized trial personnel will enter data into the electronic database.

Prior to the release into the productive environment, the electronic trial database has been tested by the data manager and one investigator. Specific definitions of data entry fields as well as range and consistency checks for entered data values will promote data quality. Furthermore, a monitoring plan (see below) is set up to ensure that the entered data values are accurate. All entered data will be reviewed and verified by an investigator and signed-off by the principal investigator prior to data export for analyses in statistical software.

The biological data derived from the encrypted samples will be stored on a secure server of the hospital. Data will only be accessible by members of the trial team.

### Confidentiality {27}

The investigators affirm and uphold the principle of the participant’s right to privacy and will comply with the applicable privacy laws. The anonymity of the participants will be guaranteed when presenting the data at scientific meetings or publishing them in scientific journals. Individual medical information obtained as a result of this trial will be considered confidential and will not be disclosed to third parties. Direct access to not encrypted source documents will be permitted for authorized third parties for purposes of monitoring, audits, and inspections. The trial staff will have access to the trial data based on authorization by the principal investigator. Confidentiality will be established by utilizing identification codes to link participants with trial data. The identification codes will not contain name, initials, date of birth, or any other personal identification numbers (e.g., social security number, patient identification number, etc.). The list with the assigned trial identification codes and minimal personal information (i.e., name, date of birth) will be stored in a locked cabinet at the trial site. All CRFs and data collection files will be identified with the trial code only. After trial closure, all trial data will be stored in a locked archive room with limited access for 10 years.

### Plans for collection, laboratory evaluation, and storage of biological specimens for genetic or molecular analysis in this trial/future use {33}

All processing of biological specimens will be done with encrypted samples (blood and urine). The samples from each assessment time point will be transferred for processing to the SwiSCI Biobank, which has been audited and certified by the Swiss Biobanking Platform (SBP). The processing of the samples will be done preferably by a team of two persons (preferably) according to validated workflows and SOPs, starting with the most perishable sample (urine), in order to ensure the shortest possible processing (needle-to-freezer) time. A biobanking software (FreezerPro Brooks, Chelmsford, MA, USA) will be used for documentation as well as sample and aliquot tracking. Samples will be accessed and withdrawn according to validated procedures and analyzed according to the trial protocol. Any unused samples will be stored for at least 5 years after the end of the trial.

## Statistical methods

### Statistical methods for primary and secondary outcomes {20a}

All the data of randomized trial participants will be analyzed blinded and according to an intention-to-treat basis. This is a feasibility trial, and thus, descriptive statistics will be used. All outcome measures will be presented as point estimates and 95% confidence intervals. Differences in clinical and laboratory outcome measures between the two treatment groups will be evaluated based on confidence intervals.

### Interim analyses {21b}

This is a feasibility trial, and thus, no interim analyses will be performed.

### Methods for additional analyses (e.g., subgroup analyses) {20b}

This is a feasibility trial, and thus, there will be no subgroup analyses.

### Methods in analysis to handle protocol non-adherence and any statistical methods to handle missing data {20c}

All available data will be included for intention-to-treat analysis. Missing data will not be imputed.

### Plans to give access to the full protocol, participant-level data, and statistical code {31c}

Access to the full protocol, participant-level data, and statistical code will be given upon reasonable request to the corresponding author.

## Oversight and monitoring

### Composition of the coordinating center and trial steering committee {5d}

There is no coordinating center or trial steering committee.

### Composition of the data monitoring committee, its role, and reporting structure {21a}

This is a mono-centric pilot trial evaluating the feasibility of a main trial investigating an approved medicinal product with a low-risk profile. Thus, there is no data monitoring committee.

### Adverse event reporting and harms {22}

Treatment side effects are collected from self-report questionnaires fortnightly during the treatment period.

All SAEs are collected and documented from the start of the trial treatment until 7 days after completing or terminating the treatment (safety follow-up time). Any suspected unexpected (not consistent with product information) serious adverse reaction (SUSAR) will be reported to the ethics committee within 15 days (events resulting in death within 7 days) of becoming aware of the event.

### Frequency and plans for auditing trial conduct {23}

An independent monitor from the CTU of the Swiss Paraplegic Centre will verify that the rights and well-being of the trial participants are protected; that the collected data are accurate, complete, and verifiable from source documents; and that the conduct of the trial complies with the currently approved protocol, the International Council for Harmonisation E6(R2) Good Clinical Practice Guideline, and the applicable regulatory requirements. Monitoring will be performed according to the monitoring plan (version 1.1 dated 18.11.2019) developed for the trial (Supplement 1).

### Plans for communicating important protocol amendments to relevant parties (e.g., trial participants, ethical committees) {25}

Substantial amendments are submitted to the ethics committee for approval before implementation. In emergency circumstances, deviations from the protocol to protect the rights, safety, and well-being of trial participants will proceed without prior approval by the ethics committee. However, these deviations and measures will be documented and reported to the ethics committee within 7 days. Active trial participants will be informed regarding all relevant protocol amendments concerning their rights, safety, and well-being.

All non-substantial amendments are communicated to the ethics committee within the annual safety report.

## Dissemination plans {31a}

The data from the present trial will be presented at scientific meetings and published in peer-reviewed journals. The authorship will be determined based on the contributions of the individuals involved.

## Discussion

Due to the lack of evidence, a plethora of measures and treatments are currently applied to prevent UTIs in individuals with NLUTD. Specific measures are chosen and combined according to personal convictions or preferences and mainly aim at preventing infections with *E. coli*, which is the most commonly detected microorganism in UTIs in these patients [19]. Oral immunomodulation with a lysate of UTI-relevant *E. coli* is thus a promising preventative measure against *E. coli* UTI, but the evidence is lacking, too. Furthermore, there are preliminary observations that immunomodulation with a *E. coli* lysate may also be beneficial in UTI caused by other bacteria [14]. However, there is a lack of data from randomized placebo-controlled trials with clinically relevant outcome measures.

Overly optimistic forecasts regarding eligibility and enrollment rates, overly strict eligibility criteria, competition with other trials, and undue patient burden are major reasons why too many trials either require more time to complete recruitment goals or fail to enroll the targeted number of participants [20]. A realistic estimation of the enrollment rate at a trial center is crucial for planning a successful trial and requires a pilot phase. In order to optimize the enrollment rate, we have established defined screening and recruitment procedures and appointed a dedicated trial coordinator. A trial coordinator helps to prioritize a trial within the busy clinical routine and will have a positive effect not only on enrollment, but also on retention of trial participants [21, 22]. In order to improve retention further, the trial protocol was drafted to be in line with the clinical processes at the trial center. Patients are more inclined to consent to trial participation if they believe that they will profit, which is not the case when they are assigned to the placebo group [23]. Thus, we make sure that patients understand they will receive standard of care in case of a UTI. Uro-Vaxom® has a good safety profile [11, 12, 15], and thus, concerns regarding side effects should not represent a major issue. Additional tests, depending on the number, invasiveness, and time requirements, increase the burden of patients and have a negative effect on enrollment and retention [20]. We have therefore minimized the burden of trial assessments. In the present trial, potential participants are approached within the first 56 days after SCI, when many still feel overwhelmed by the new life-changing condition and thus may rather decline participation than consent [24]. Nevertheless, we have chosen this population to investigate, whether immunomodulation has an effect during the phase of immuno-depression shortly after SCI [25] and whether it prevents bladder colonization with pathogenic bacteria, which is very common in this population. Furthermore, running a trial during primary rehabilitation will improve adhesion to trial medication [26] and eliminate the need to return to the trial center for follow-up evaluations. We paid special attention to the information and instruction of the nursing and medical staff, in order to ensure administration of the trial drug and reporting of SAEs according to the trial protocol. The trial is embedded within a cohort study and thus benefits from established screening and sampling processes. However, the requirement to first consent to the participation in the cohort study will preclude some patients from enrolling in the trial. Apart from enrollment, retention, and adhesion rates, this pilot trial will also assess other feasibility issues like costs and adequate staffing.

The primary outcome of the future main trial will be the count of symptomatic UTI during the observation period. The diagnosis of a clinically relevant UTI in the chosen population is challenging as a result of the mediocre reliability of patient-reported UTI symptoms [27] and the very common presence of bacteriuria [24]. Furthermore, counting symptomatic UTI requiring antibiotic treatment in an out-patient setting is affected by the relatively subjective assessment of involved clinicians. The current trial runs in an in-patient setting, and thus, urine analysis and culture results will be available to define the occurrence of a UTI.

The unique feature of the present trial is the objective to evaluate not only the clinical (number of symptomatic UTI) effects of immunomodulation, but furthermore to investigate the biochemical and cellular responses of the immune system and the effects on the urinary microbiome. We will investigate whether immunomodulation with Uro-Vaxom® has the potential to stimulate a weakened immune system shortly after SCI, as shown in an animal study [28], and to prevent or decrease bladder colonization with pathogenic bacteria.

The concealment of group allocation (blinding) from trial participants and staff is a critical methodologic feature of a randomized controlled trial. In order to minimize the costs and administrative hurdles in this pilot trial, we have chosen not to blind the trial participants. Trial participants will not be informed regarding treatment allocation. However, the trial personnel and the biostatistician will be blinded regarding treatment allocation. Standard clinical treatment of UTIs will be permitted. It would be unethical to withhold antibiotic treatment of acute symptomatic UTIs from trial participants. Antibiotics will have an effect on the urinary microbiome and thus represent a limitation of the present trial.

## Trial status

Protocol version 1.3 dated 10.07.2020. Recruitment began on June 1, 2020, and is expected to be complete by May 31, 2023.

## Supplementary Information

**Additional file 1:.** Monitoring plan

## Abbreviations

AIS: American Spinal Cord Injury Association impairment scale
CFU: Colony-forming units
CRF: Case report form
CTU: Clinical Trial Unit
DNA: Deoxyribonucleic acid
ELISA: Enzyme-linked immunosorbent assay
*E. coli*: *Escherichia coli*
eCRF: Electronic case report form
IFN: Interferon
Ig: Immunoglobulin
IL: Interleukin
NLUTD: Neurogenic lower urinary tract dysfunction
pCRF: Paper case report form
RNA: Ribonucleic acid
RT-PCR: Reverse transcription polymerase chain reaction
SAE: Serious adverse event
SCI: Spinal cord injury
SBP: Swiss Biobanking Platform
SOP: Standard operating procedure
SUSAR: Suspected unexpected serious adverse reaction
SwiSCI: Swiss Spinal Cord Injury Cohort Study
UTI: Urinary tract infection
TGF: Tumor growth factor
TNF: Tumor necrosis factor
