# Supplementary material for: Immunomodulation for primary prevention of urinary tract infections in patients with spinal cord injury during primary rehabilitation: protocol for a randomized placebo-controlled pilot trial (UROVAXOM-pilot)
Source: Trials. 2021 Oct 4;22:677. doi: 10.1186/s13063-021-05630-w (PMC8489057; doi:10.1186/s13063-021-05630-w)
Supplement: Supplementary file 1 — Additional file 1:. Monitoring plan [file 13063_2021_5630_MOESM1_ESM.docx]

Monitoring Plan

**Study Details**

| **Study Title** | Immunomodulation therapy for primary prevention of urinary tract infections in patients with spinal cord injury during first rehabilitation: a randomized controlled pilot study |
| --- | --- |
| **Short Title** | Immunomodulation therapy for urinary tract infections |
| **Study Identifier** | UROVAXOM-Pilot |
| **Study Number** | 2018-22 |
| **Trial Registration** | NCT04049994 |
| **Protocol Version and Date** | Version 1.1 / 18.11.2019 |
| **Study Type / Category** | Interventional study with investigational medicinal product (IMP) / risk category A |
| **Investigational Product** | UroVaxom: lyophilized lysate of 18 Escherichia coli strains |
| **Study Center(s)** | Single-center: Neurourology, Swiss Paraplegic Centre, Nottwil |
| **Sponsor-Investigator** | Jürgen Pannek, Prof. Dr. med. |
| **Contact Person for Monitoring** | Jörg Krebs, Dr. med. vet., PhD |
| **Monitor** | Monitor Clinical Trial Unit Swiss Paraplegic Centre Guido A. Zäch Strasse 4 6207 Nottwil |

**Monitoring Plan**

| Version / Date of Monitoring Plan | Version 1.1 / 18.11.2019 |
| --- | --- |
| Author of Monitoring Plan | Monitor Clinical Trial Unit, Swiss Paraplegic Centre |

**Monitoring category based on risk-adapted approach**

Details and extent of monitoring are defined by the risk category according to the Human Research Act and adapted by the riskADApted MONitoring (ADAMON) analysis. Site specific risk factors can increase the extend of monitoring for a study.

| Risk-adapted Monitoring Class  according to ADAMON | Low risk – category K3 |
| --- | --- |
| Risk score  based on evaluation of further criteria | Risk is confirmed |

**Monitoring Visits**

| **Visit** | **Schedule** | **Comments** |
| --- | --- | --- |
| Site initiation visit | prior to the start of the study |  |
| Monitoring visit 1 | after inclusion of first study participant |  |
| Monitoring visit 2 | after study completion of first participant |  |
| Monitoring visits 3 + n | annually after inclusion of first participant | additional visits on Sponsor's request or in case of major or critical findings |

**Scope and Extent of Monitoring**

| **Action** | **Extent (%)** | **Comments** |
| --- | --- | --- |
| Existence of patients | 100% |  |
| Informed consent | 100% |  |
| Inclusion/exclusion | 20% | in case of major or critical findings: 50% |
| Primary Endpoint | 20% | in case of major or critical findings: 50% |
| Safety (AE/SAE) | 100% |  |
| Drug accountability | 20% | in case of major or critical findings: 50% |
| Source Data Verification (SDV) | 1 participant 100% | in case of major or critical findings: 2 participants |

Furthermore, the following documents of the trial master file will be reviewed:

- ethics approval
- study protocol version
- screening and enrollment log, patient identification log
- randomization process
- informed consent form version
- delegation log, staff list
- source documents (source data location log)

**Monitoring Report**

The monitor will document the findings in the Monitoring Report and discuss the findings with the Investigator. The Sponsor will receive the Monitoring Report within 7 working days.
